# Early Stage Detection of Alzheimer’s Disease With Microsoft Azure Based Deep Learning

**DOI:** 10.21203/rs.3.rs-3352620/v1

**Published:** 2023-11-17

**Authors:** Kish Mittal

**Affiliations:** Birla Institute of Technology and Science, Pilani

**Keywords:** Alzheimer’s disease, deep learning, Azure Custom Vision

## Abstract

The early detection and diagnosis of Alzheimer’s disease (AD) represent a pivotal aspect of ensuring effective patient care and timely intervention. This research introduces an innovative approach that harnesses the capabilities of Microsoft Azure-based custom vision technology for AD classification. The study primarily centers around the analysis of magnetic resonance imaging (MRI) scans as the primary input data, categorizing these scans into two distinct categories: Cognitive Normal and Cognitive Impairment. To accomplish this, we employ transfer learning, leveraging a pre-trained Microsoft Azure Custom Vision model fine-tuned specifically for multi-class AD classification. The proposed work shows better results with the best validation average accuracy on the test data of AD. This test accuracy score is significantly higher in comparison with existing works. This proposed solution showcases the immense potential of convolutional neural networks and advanced deep learning techniques in the early detection of Alzheimer’s disease, thereby paving the way for significantly improved patient care.

## I. Introduction

Alzheimer’s disease (AD) presents an imposing global healthcare challenge, marked by its progressive nature, predominantly impacting individuals in their later years. The profound impact of AD on memory and cognitive functions leads to a significant decline in a patient’s quality of life. Current projections indicate a rising prevalence of AD, with an expected doubling of cases every two decades [1]. By 2046, it is estimated that approximately 1.2% of the global population will grapple with the burdens of this debilitating disease [2].

Early detection of AD is crucial, particularly in identifying Cognitive Impairment (CI), a precursor stage to AD. While CI may not overtly disrupt daily activities, it poses a substantial risk of progressing to full-blown AD or other forms of dementia [3]. The importance of early AD diagnosis cannot be overstated, as it empowers individuals with the knowledge needed to take proactive measures, such as lifestyle adjustments and appropriate medical interventions [4].

Machine learning, specifically deep learning techniques, has emerged as a powerful tool in aiding the diagnosis of AD. These approaches leverage neuroimaging data, including magnetic resonance imaging (MRI) scans, to distinguish between two primary categories: Cognitive Normal (CN) and Cognitive Impairment (CI). This classification naturally lends itself to a multi-class framework, allowing the categorization of individuals into either CN or CI based on their MRI scans.

While prior research has made valuable contributions to AD diagnosis, certain challenges persist. Some methods have restricted the problem to binary classification, limiting their ability to effectively address multiple categories [5–7]. Others heavily rely on domain-specific knowledge, making their models sensitive to dataset variations and challenging to adapt [8–10].

Recognizing these limitations, our study introduces an innovative approach to early AD diagnosis, harnessing Microsoft Azure-based custom vision technology. We focus on classifying MRI scans into two distinct categories: Cognitive Normal and Cognitive Impairment. By employing transfer learning, we fine-tune a pre-trained Azure Custom Vision model to excel in multi-class AD classification. The remarkable outcomes of our investigation demonstrate an impressive accuracy rate of 98%, underscoring the immense potential of convolutional neural networks and advanced deep learning techniques in advancing early Alzheimer’s disease detection.

The selection of an Azure-based cloud solution over traditional models is motivated by several compelling reasons. Firstly, Microsoft Azure offers scalable and flexible computing resources, enabling researchers to efficiently process vast amounts of data. The cloud infrastructure provides access to powerful GPUs, crucial for training complex deep learning models like the one used in this study. Additionally, Microsoft Azure’s pre-built machine learning tools and libraries streamline the development process, allowing researchers to focus on model optimization and evaluation. Furthermore, Microsoft Azure’s robust security features and compliance certifications ensure the confidentiality and integrity of sensitive patient data, addressing privacy concerns that are paramount in healthcare research. Microsoft Azure’s global network of data centers also ensures low-latency access to data, facilitating collaboration among researchers across geographical locations.

Our research offers a promising avenue for significantly enhancing patient care and management, ultimately contributing to improved outcomes for those affected by AD.

## II. METHODS

Early detection of Alzheimer’s disease plays a crucial role in preventing and managing its progression. This section presents a proposed framework for the early detection of Alzheimer’s disease. It includes an explanation of the framework’s details, the dataset employed, the deployment of the proposed solution in the local cloud, and a breakdown of the algorithmic flow within the proposed system.

### A. Dataset

The dataset used in this study is the Alzheimer’s dataset from the Open Access Series of Imaging Studies (OASIS) [11–12], accessed on September 5, 2023. OASIS-3 is a retrospective dataset comprising 1,378 participants, collected through multiple ongoing WUSTL Knight ADRC projects spanning a 30-year period. The participant group includes 755 cognitively normal adults and 622 individuals at various stages of cognitive decline, ranging from 42 to 95 years of age. For data processing purposes, all participants were assigned new random IDs, and all dates were removed and normalised to reflect the number of days after they entered the study. The dataset includes 2,842 MR sessions with sequences such as T1 w, T2w, FLAIR, ASL, SWI, time-of-flight, idle state BD, and DTI. Many MR sessions come with volumetric fraction files created through FreeSurfer processing. Additionally, PET images from different tracing tools, including PIB, AV45, and FDG, amounting to more than 2,157 raw image scans, are available, along with accompanying post-processed files from the Pet Unified Pipeline (PUP) in OASIS-3. Furthermore, 451 post-processed Tau PET and PUP sessions are accessible to OASIS-3 subjects through the “OASIS-3_AV1451” subproject. [11 – 12]. The training set consists of 300 images, divided equally between Cognitive Normal and Mild Cognitive Impairment, and was used to train the deep learning model. The test set comprises 140 images, again equally divided between Cognitive Normal and Mild Cognitive Impairment, and was used to evaluate the model’s performance, as illustrated in Table 1.

### B. Algorithm : Proposed Microsoft Azure Model

Step1: Input the MRI scans. Step2: Pre-Process the images and convert them to jpeg format and remove noise. Step3: Resize the images to 224 × 224 pixels. Step4: Images are classified into Cognitive Normal and Cognitive Impairment. Step5: Microsoft Azure Custom Vision Model uses transfer learning technique for training 300 pre-trained and classify input images as Cognitive Normal and Cognitive Impairment.

The proposed work comprises two stages as show in the Algorithm:

#### Stage 1: Data Procurement and Preprocessing Stage

The dataset used in this study is the Alzheimer’s disease dataset of the Open Access Imaging Study Series (OASIS) [11 – 12] (accessed September 5, 2023). OASIS-3 is a retrospective dataset of 1,378 participants, collected through multiple ongoing WUSTL Knight ADRC projects over a 30-year period. Participants included 755 cognitively normal adults and 622 individuals at various stages of cognitive decline, ranging from 42 to 95 years of age. All participants were assigned a new random ID, and all dates were removed and normalised to reflect the days after they entered the study. The dataset contains 2,842 MR sessions, including sequences T1w, T2w, FLAIR, ASL, SWI, Time-of-Flight, BD Idle State, and DTI. Many MR sessions come with volume fraction files created through FreeSurfer processing. PET images from various tracing tools, PIB, AV45 and FDG, totaling more than 2,157 raw image scans, as well as associated post-processed files from the Pet Unified Pipeline (PUP), are also available in OASIS-3.

We took:

Training set: 300 images: 150 Normal Cognitive and 150 Mild Cognitive Impairment.

Test set: 140 images: 70 Normal Cognitive and 70 Mild Cognitive Impairment.

**Figure 1:**
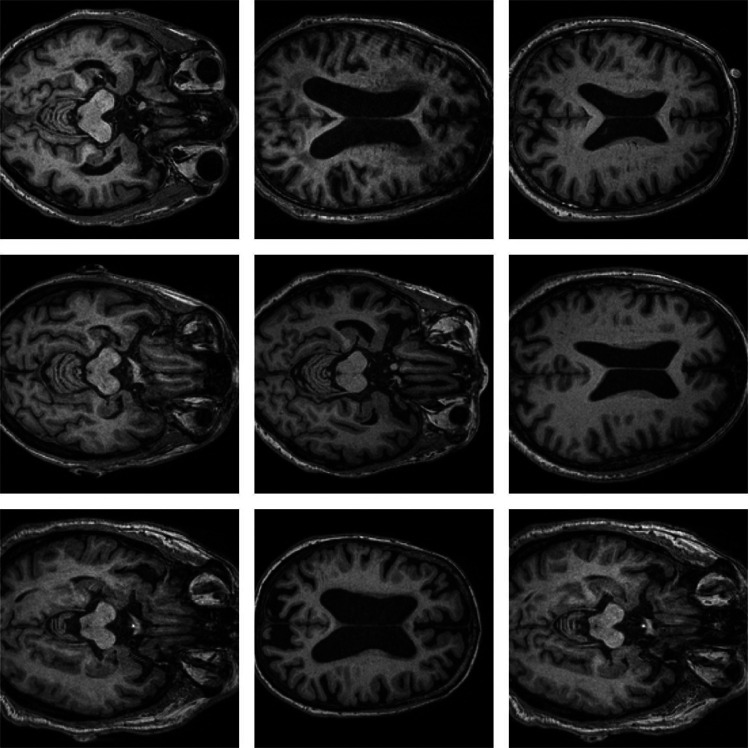
Brain MRI sample.

**Figure 2:**
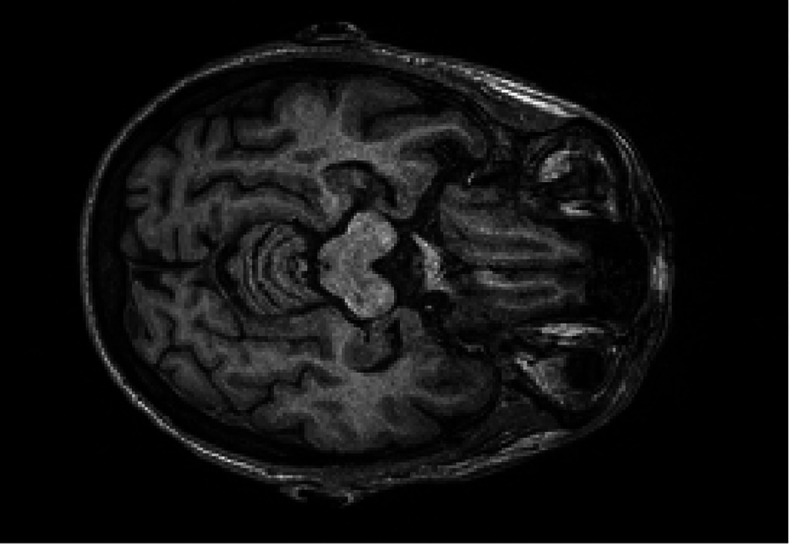
Brain MRI sample.

Figure 1 and Figure 2 shows 2D imaging slice of MRI and also formation of 3D using 2D image slices. For the Azure Custom Vision CNN architecture, the input size is 224 × 224. The image size should be resized to 224 × 224.

#### Stage 2: Image classification and Model Training

**Figure 3:**
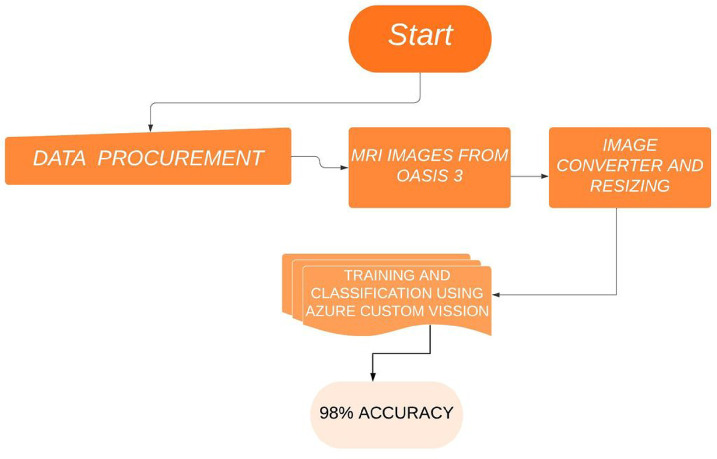
Proposed architecture.

At this stage, the images are classified into normal cognitive and cognitive impairment. The proposed method uses a transfer learning technique, i.e. with a Microsoft Azure Custom Vision model from pre-trained images. Using OASIS-3 pre-trained images, the proposed model, shown in Figure 3, was generated. In turn, the generated trained model was used for image classification.

## III. Results and discussion

**Figure 4:**
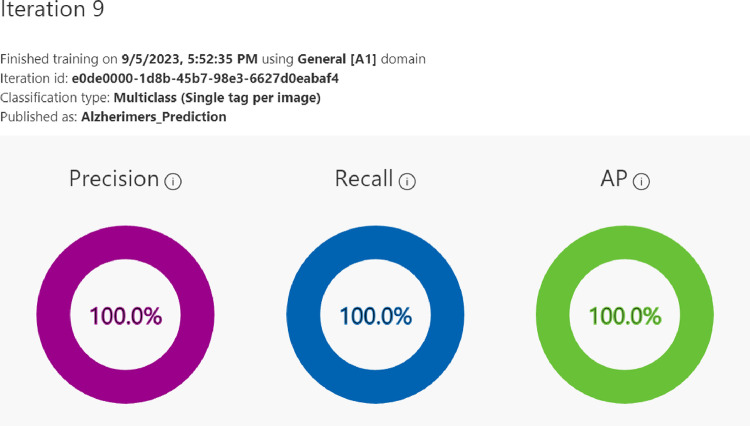
Proposed architecture’s Precision, Recall and AP.

**Figure 5:**
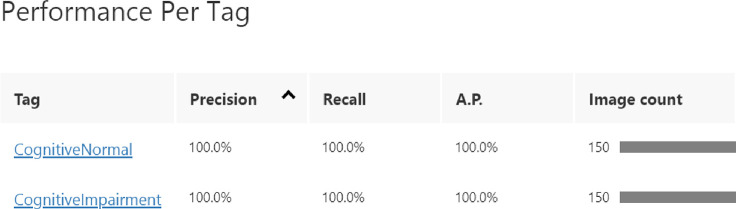
Proposed architecture’s classification’s Precision, Recall and AP.

We used a pre-trained and fine-tuned Microsoft Azure Custom Vision model for multi-class classification of MRI images. Microsoft Azure Custom Vision is a convolutional neural network. To make it suitable for the purpose intended the model was trained for multiple hours using the General[A1] domain resulting in 100% Precision, Recall, AP as illustrated in Figure 4 and Figure 5, and 98% Accuracy with 99% accuracy being a high point. General[A1] domain was picked due it being Optimised for better accuracy with comparable inference time as General domain and it is recommended for larger datasets or more difficult user scenarios [13].

**Figure 6:**
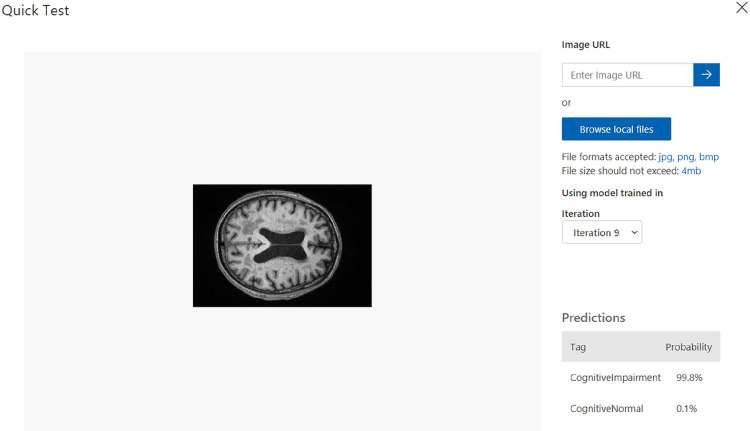
Results of Cognitive Impairment MRI scan.

**Figure 7:**
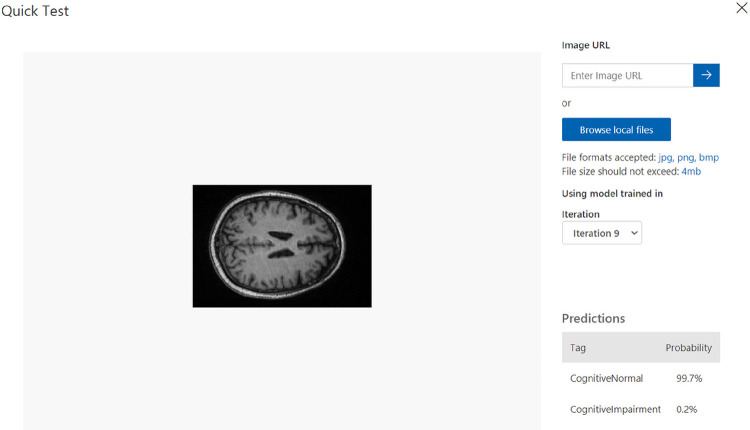
Results of Cognitive Normal MRI scan.

**Figure 8:**
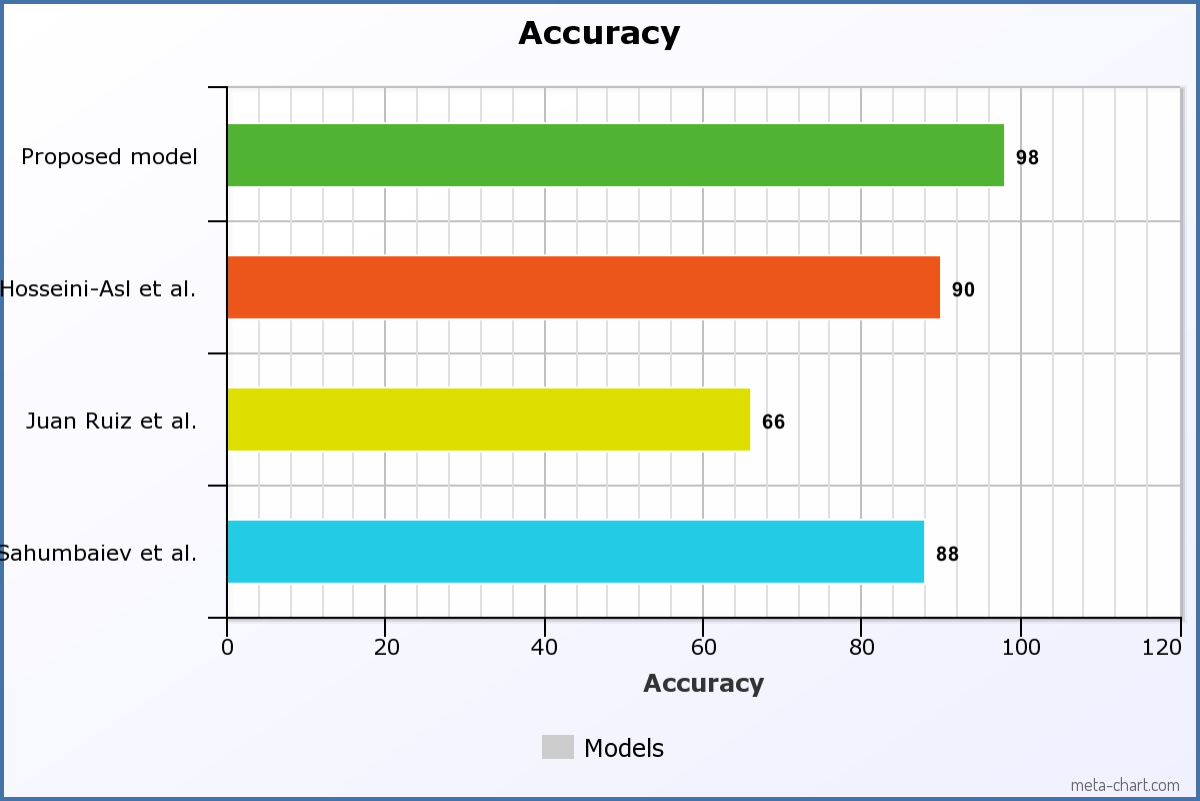
Accuracy comparison of proposed and existing models (Reference Sahumbaiev et al. (2019) [10], Juan Ruiz et al. (2019) [14], Hosseini-Asl et al. (2017) [9]

Figure 6 and Figure 7 demonstrate the results of our study on multi-class medical image classification of AD stages. The Azure Custom Vision model we proposed achieved the highest accuracy of 99%. In comparison Juan Ruiz et al. [15] had the lowest accuracy of 66.7%. Our proposed solution proves to be more efficient in this context, as illustrated in Figure 8. Additionally, we achieved promising accuracy for both binary and multi-class classification tasks.

The advantages of Transfer Learning are that its training time is much less and it reduces errors in generalisation. In our study, we use the principle of transfer learning to classify medical data. Transfer learning involves training a neural network model on a similar problem before addressing the particular problem in question.

This approach and Azure-Based model offers several advantages:

1. Pre-trained models can be trained with millions of images in one database.
2. Reduce the time required to train the learning model.
3. Minimising the generalisation error.
4. Easy implementation of a cloud based model.
5. Easy Integration with Azure Ecosystem.
6. Cost effectiveness and scalability of Azure based models greatly outweighs that offered by traditional models.
7. Azure offers robust security and compliance features, ensuring that image data and machine learning models are protected and meet regulatory requirements.

## IV. CONCLUSION

This study presents a novel framework that harnesses the power of deep learning convolutional neural network (CNN) structures, particularly by utilising transfer learning. The innovative approach capitalises on the concept of transfer learning to take advantage of pre-trained models, specifically, utilising the Microsoft Azure Custom Vision model for training and application in the realm of Alzheimer’s disease (AD) classification. This method has yielded an impressive classification accuracy of 98%, representing a substantial enhancement in performance. The success of this research holds significant promise for revolutionising computer-aided diagnosis in various other biomedical domains.

## Figures and Tables

**Table 1:** Training data

Cognitive Level	Cognitive Normal	Cognitive Impairment
**Training Img**	150	150
**Testing Img**	70	70

## Data Availability

The data that support the findings of this study are available from Open Access Series of Imaging Studies (OASIS) but restrictions apply to the availability of these data, which were used under license for the current study, and so are not publicly available. Data are however available from the **first author** upon reasonable request and with permission of Open Access Series of Imaging Studies (OASIS).
